# Effect of Bilayer Thickness and Bias Potential on the Structure and Properties of (TiZr/Nb)N Multilayer Coatings as a Result of Arc-PVD Deposition

**DOI:** 10.3390/ma15217696

**Published:** 2022-11-01

**Authors:** Rakhadilov Bauyrzhan, Pogrebnjak Alexander, Sagdoldina Zhuldyz, Buitkenov Dastan, Beresnev Vyacheslav, Amina Mukhamedova

**Affiliations:** 1 Surface Engineering and Tribology Research Center, Sarsen Amanzholov East Kazakhstan University, Ust-Kamenogorsk 070002, Kazakhstan; 2 Department of Nanoelectronics and Surface Modification, Sumy State University, Rymskogo-Korsakova Str., 40007 Sumy, Ukraine; 3 Department of Motor Vehicles, Lublin University of Technology, Nadbystrzycka 38D, 20-618 Lublin, Poland; 4 Department of Materials for Reactor Building and Physical Technologies, V.N. Karazin Kharkov National University, Svobody 4, 61000 Kharkov, Ukraine; 5 Faculty of Basic Engineering Training, D. Serikbayev East Kazakhstan Technical University, Ust-Kamenogorsk 070000, Kazakhstan

**Keywords:** multilayer coating, Arc-PVD, (TiZr/Nb)N, phase, wear resistance, hardness

## Abstract

This work is devoted to the study of the formation of nanostructured multilayer coatings (TiZr/Nb)N on the surface of an AISI 321 steel substrate depending on the deposition parameters of the Arc-PVD method. The results of the X-ray diffraction analysis showed the formation of solid solution (TiNb)N and ZrN in the multilayer coatings with an FCC structure, ε-NbN with a hexagonal structure, as well as with a small volume fraction of the ε-Ti_2_N and β-Nb_2_N phase. On the basis of phase composition data, it is possible to assume that an increase in the number of bilayers leads to a decrease in the nitrogen concentration in the bilayers and, consequently, to a decrease in the volume fraction of ε-NbN and β-Nb_2_N nitrides. In all investigated systems obtained at −100 V and −200 V bias potentials, ε-NbN is the main phase. The study of the element distribution over the thickness of the (TiZr/Nb)N coating confirms the results of the X-ray diffraction analysis. The use of the structure model in the form of alternating layers allows for significantly improving the adhesion characteristics of the protective coating, as well as ensuring their high hardness. Based on the experimental results, it is possible to analyze changes in the mechanical and tribological properties of multilayer coatings depending on the number of applied bilayers. The results of the study of the elastic modulus and hardness of multilayer coatings (TiZrNb)N with different numbers of bilayers showed that a large number of bilayers (small thickness of each individual layer) shows the lowest value of hardness. It is assumed that as the bilayer thickness decreases, the coating characteristics are closer to the monolayer alloy than to the multilayer structure.

## 1. Introduction

Coatings of solid transition metal nitrides deposited by physical vapor deposition (PVD) are successfully used in industry. Especially, CrN, TiN, ZrN, and NbN coatings have shown remarkable corrosion and wear protection properties and played an important role a few years ago due to their increased hardness and strength, as well as a lower coefficient of friction compared to high-speed steel [1,2,3,4,5,6].

Recently, increasing attention has been paid to nitride coatings with multilayer architecture due to their high mechanical and tribological characteristics [7,8,9,10,11,12,13,14]. The multilayer architecture of the obtained coatings makes it possible to influence the structural-phase state of the layers by changing the modulation period of alternating layers, and, in addition, allows for combining their individual properties [15,16,17,18].

The alternation of two layers that have different properties makes it possible to create a coating with unique characteristics [19,20,21,22,23,24]. A change in the period of the bilayer, in turn, affects the state of the interfaces between the deposited materials, which play an important role in the properties of the coatings [3,6,25,26,27]. The bilayer period is one of the most important parameters in multilayer coatings since the ultimate hardness usually occurs in a narrow range (5–15 nm) [28,29]. Several explanations were proposed, including blocking of dislocations by layer boundaries, Hall–Petch hardening, deformation effects at layer boundaries, and the effect of super hardness [30]. Reducing the thickness of the bilayer down to the nanoscale at a constant value of the total thickness of the coating makes it possible to increase the number of interfaces between the layers, which act as a barrier to dislocation migration and the propagation of microcracks [31,32]. A deeper understanding of this issue is the basis for the subsequent development of functional multilayer coatings.

In addition, multilayer coatings consisting of transition metal nitrides are currently under the scrutiny of researchers due to their advantageous properties that satisfy many industrial requirements, as well as because of their special scientific significance [33]. Therefore, the preparation and study of previously unstudied (TiZr/Nb)N multilayer coatings are of great interest. The main goal of this research is to evaluate the effect of chamber pressure and the number of bilayers and/or the period of bilayers on (TiZr/Nb)N coatings deposited on AISI 321 (08Kh18N9T) steel substrates by the Arc-PVD method in relation to their mechanical and tribological characteristics.

## 2. Materials, Equipment and Research Methods

**Figure 1:**
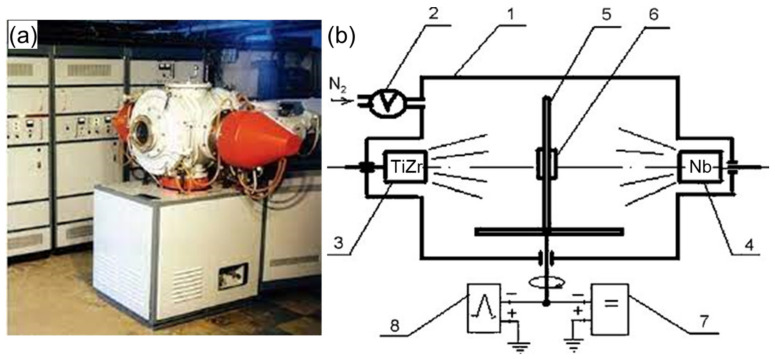
Vacuum arc device “Bulat-6” (**a**) outside view of device; (**b**) schematic representation of deposition system: 1—acuum chamber; 2—automatic system of nitrogen pressure control; 3,4—TiZr, Nb evaporators; 5—substrate holder; 6—substrate; 7—DC voltage source; 8—high-voltage pulse generator.

Multilayered coatings were produced by the cathodic arc physical vapor deposition (Arc-PVD) by means of vacuum arc device Bulat-6 (Figure 1) [2,34]. AISI 321 steel was used as a substrate. Material composition was Ti, 75at.%; Zr, 25at.%. and Nb (99.84%). The deposition was carried out in a nitrogen atmosphere from two sources TiZr and Nb, respectively, with a fixed substrate stopping time of 10, 20 or 40 s at each of the two vaporized sources. Multilayer coatings (TiZr/Nb)N with different numbers of bilayers were obtained under the modes presented in Table 1.

X-ray phase studies of the samples were performed by X-ray diffraction analysis on an X’PertPro (“PANalytical”, Almelo, The Netherlands) diffractometer. X-ray diffraction patterns were taken using CuKα radiation at a voltage of 40 kV and a current of 30 mA. The X-ray diffraction patterns were interpreted using the ICDD PDF-2 database. Analysis of the elemental composition, aimed at revealing the distribution of components over the depth of the sample, was carried out using a Shkhuna-2 Auger electron spectrometer.

The mechanical properties (hardness, Young’s modulus) of the coatings were studied on the NanoScan-4D Compact (FSBI TISNCM, Moscow, Russia) nanohardness tester. The tests were carried out at a load of 100 mN. Loading time—5 s, unloading time—5 s, maximum load holding time—5 s. The dependence of the penetration depth on the applied force at the stages of loading and unloading was determined by the Oliver–Pharr method [35]. At least 10 measurements were carried out on each sample, the results of which were averaged. The CSEM Micro Scratch Tester (Neuchatel, Switzerland) was used to study the adhesion characteristics of coatings using the “scratch” method. Scratch testing was carried out at a maximum load of 30 N, the rate of change in the normal load on the sample was 29.99 N/min, the indenter speed was 6.794 mm/min, the scratch length was 7 mm, and the radius of the tip was 100 μm. To obtain reliable results, three scratches were applied to the surface of each coated sample.

**Figure 2:**
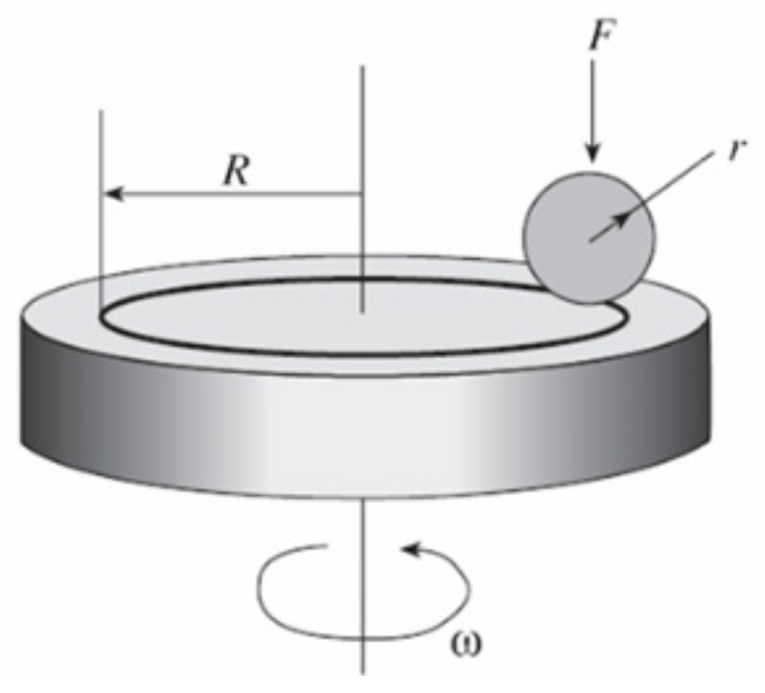
Tribological tests of samples according to the “ball-and-disk” scheme.

Tribological tests were carried out on a TRB^3^ (Anton Paar Srl, Peseux, Switzerland) tribometer using the standard ball-and-disk (Figure 2) technique (international standards ASTM G 133-95 and ASTM G99) [36,37]. A ball 3 mm in diameter made of ShKh15 (AISI 52100) steel was used as a counter specimen [16]. The tests were carried out at a load of 5 N and a linear velocity of 15 cm/s, a wear curvature radius of 5 mm, a friction path of 600 m. The profilograms of the friction surface of the coatings and volumetric wear losses of the coatings were determined using a non-contact 3D profilometer MICROMEASURE 3D (STIL, France) station. The obtained profiles were analyzed using the Mountains Map Universal v.2.0.13 software.

## 3. Experimental Results and Discussion

**Figure 3:**
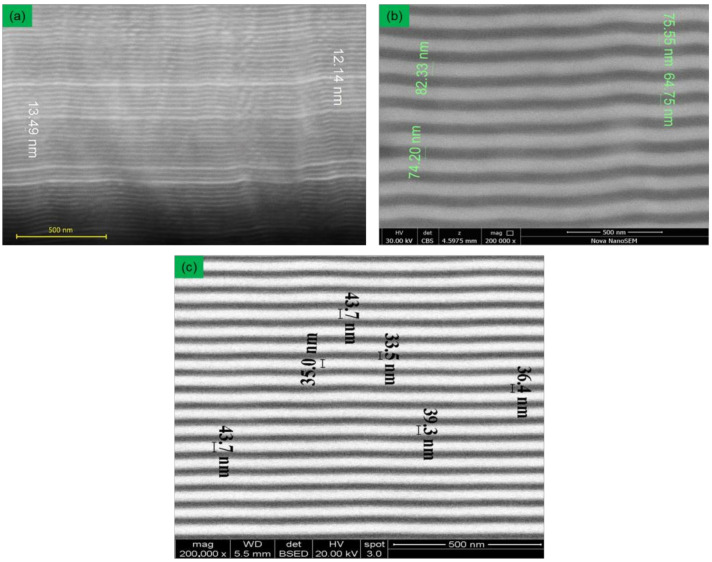
Cross-section images of multilayer (TiZr)N/NbN coatings: (**a**) deposition time of 10 s; (**b**) deposition time of 40 s; (**c**) deposition time of 20 s.

Figure 3 shows the cross-sectional images of the (TiZr)N/NbN multilayer coatings obtained by scanning electron microscopy. These images were used to determine the thickness of the layers deposited at different deposition times. It can be seen that good planarity of the layers is observed for all deposition conditions. The thickness of the layers for a single-layer deposition time of 10 s is about 12–13 nm (Figure 3a), for a single-layer deposition time of 20 s it is about 37–43 nm (Figure 3c) and for a layer deposition time of 40 s it is about 76 nm (Figure 3b). The lower deposition rate in coatings with the thinnest layers can be explained by the high specific volume of mixed layers and a larger relative error in determining the deposition time of the layer. Therefore, it is correct to carry out the most accurate determination of deposition velocity on thick layers. The deposition velocity thus obtained is about 1.8 nm/s.

**Figure 4:**
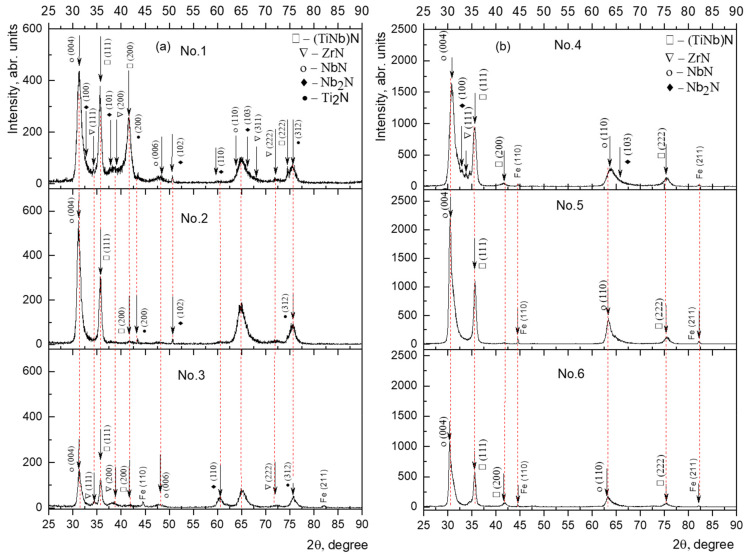
X-ray diffraction pattern of (TiZr/Nb)N coatings at a constant bias potential, U_bp_, applied to the substrate: (**a**) 200 V, (**b**) 100 V.

The results of the X-ray analysis performed in the θ/2θ geometry for the multilayer (TiZr/Nb)N films obtained at a bias potential U_bp_ = −200 V and reaction gas pressure of *p* = 1 × 10^−3^ Torr, as well as at U_bp_ = −100 V and *p* = 4 × 10^−3^ Torr, are shown in Figure 4. According to Table 2, three series of (TiZr/Nb)N coatings were obtained, which differed in the number of bilayers, consisting of 536, 270 and 134 bilayers obtained at the deposition time for each individual layer for 10 s, 20 s and 40 s, respectively.

The results of the X-ray phase analysis showed the formation of a (TiNb)N solid solution (PDF-2 2-1159, PDF-2 38-1155) and a ZrN (PDF-2 2-956) with an FCC structure, ε-NbN with a hexagonal structure (PDF-2 6-719), as well as with a small volume fraction of the ε-Ti_2_N phase (PDF-2 23-1455) and β-Nb_2_N (PDF-2 20-802) in all series of coatings. X-ray analysis of the sizes of coherent scattering regions (CSR) and internal elastic stresses (Δd/d) was carried out using the Powder Cell 2.4 full-profile analysis program. Table 2 presents the average size of the CSR and the values of micro distortions of the phase lattice obtained under different conditions of deposition of the (TiZr/Nb)N coatings. The calculation was carried out for all lines of the phase.

In all systems obtained at a bias potential of −100 V and −200 V, the main phase is ε-NbN. It is reported in [38,39] that an increase in the bias potential to U = −100 V leads to the formation of ε-NbN and the diffraction lines ε-NbN(004) and ε-NbN(110) are characteristic of NbN coatings.

The face-centered structure (FCC), very close to that of TiN, was determined for all films based on three diffraction peaks in the (111), (220), and (222) planes. Nb atoms dissolve in the TiN lattice to form a substitutional solid solution of (TiNb)N. Hume–Rothery’s basic rule states that the isomorphism of elements at temperatures far from the melting point manifests itself when the difference in atomic diameters is not more than 15% (atomic radius: titanium 0.147 nm; niobium 0.146 nm; zirconium 0.160 nm), as well as for isomorphic transformation, an important role is played by the structural factor, which is possible only if the components have a similar structure. Based on this, the formation of a substitutional solid solution of (TiNb)N is more advantageous than the formation of a (TiZr)N solid solution. Since the radius of the Zr atom is larger than that of the Ti and Nb atoms, the diffraction peaks are subject to lattice distortion. Due to the complex structure of the samples, the diffraction pattern contains many diffraction planes for different phases and some peaks overlap.

With an increase in the number of bilayers, respectively, a decrease in their thickness, and an increase in the intensity of the peaks of the (111) and (200) planes were observed compared to samples of series 2 and 3 (Figure 4a, sample No. 1), as well as a decrease in the intensity of the (004) line of the ε-NbN phase with superposition of the diffraction peak (100) of the β-Nb_2_N phase. According to [40,41], ε-NbN nitride has a region of homogeneity in the range of nitrogen concentrations of 48.0–50.6 at.%, and the β-Nb_2_N phase with a crystal lattice belonging to the hexagonal part exists in the nitrogen concentration range of 28.6–34.4 at.%. Based on the phase composition data, it can be assumed that an increase in the number of bilayers leads to a decrease in the nitrogen concentration in the bilayers and, as a consequence, to a decrease in the volume fraction of the ε-NbN and β-Nb_2_N nitrides. As the number of bilayers increases, the intensity of diffraction lines (111) and (200) of the (TiNb)N phase increases. Particularly, the increase in the number of bilayers in the coatings is supported by a progressive and intense increase in the (200) peak. The formation of the direction of phase growth in the process of the coating deposition is determined by the lowest free energy; thus, as the nitrogen concentration approaches a decrease, the (200) orientation turns out to be the most favorable. An increase in the number of alternating layers (10 s) leads to the appearance of a weak peak of the (TiZr)N solid solution, which indicates the presence of a larger proportion of interlayer transition zones in the condensate volume (Figure 4, samples No. 1 and 4).

With a further increase in the deposition time of each individual layer by a factor of two (20 s), the intensity of the ε-NbN diffraction peak with the preferred orientation (004) increases and the broadening of the diffraction peaks decreases (Figure 4b). The intensity of the ε-NbN diffraction peak depends on the bilayer thickness, i.e., the lowest intensity corresponds to the longest layer deposition period (40 s), respectively, to the highest thickness of the bilayers (Figure 4a, sample No. 3). At the deposition time of each layer of 40 s, a significant decrease in the intensity of diffraction peaks is observed and reflections from α-Fe (PDF-2 3-1050) of the steel substrate are recorded. X-ray studies have shown that the phase composition of the (TiZr/Nb)N coatings deposited under different deposition modes differ greatly in the intensity of diffraction lines; however, no significant difference in the phase composition was found. X-ray diffraction patterns of the (TiZr/Nb)N multilayer coatings formed at bias voltages on substrates of −200 V and −100 V show different ratios of the (111) and (200) diffraction peaks, which depend on the level of the electric potential applied to the substrate (Figure 4).

**Figure 5:**
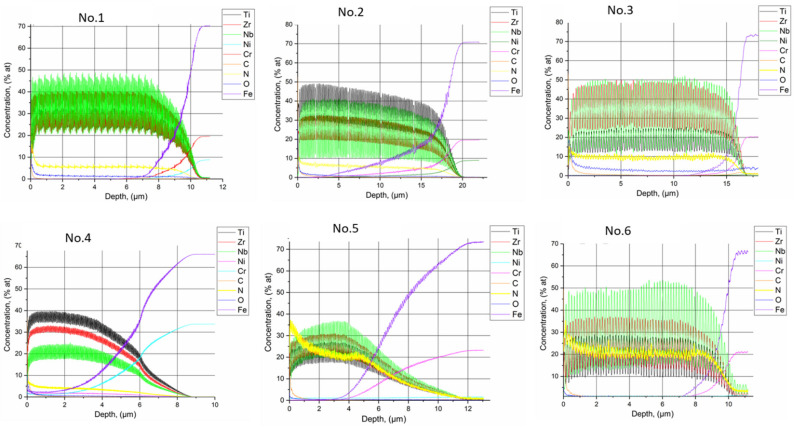
Distribution of elements over the thickness of (TiZrNb)N coatings.

**Figure 6:**
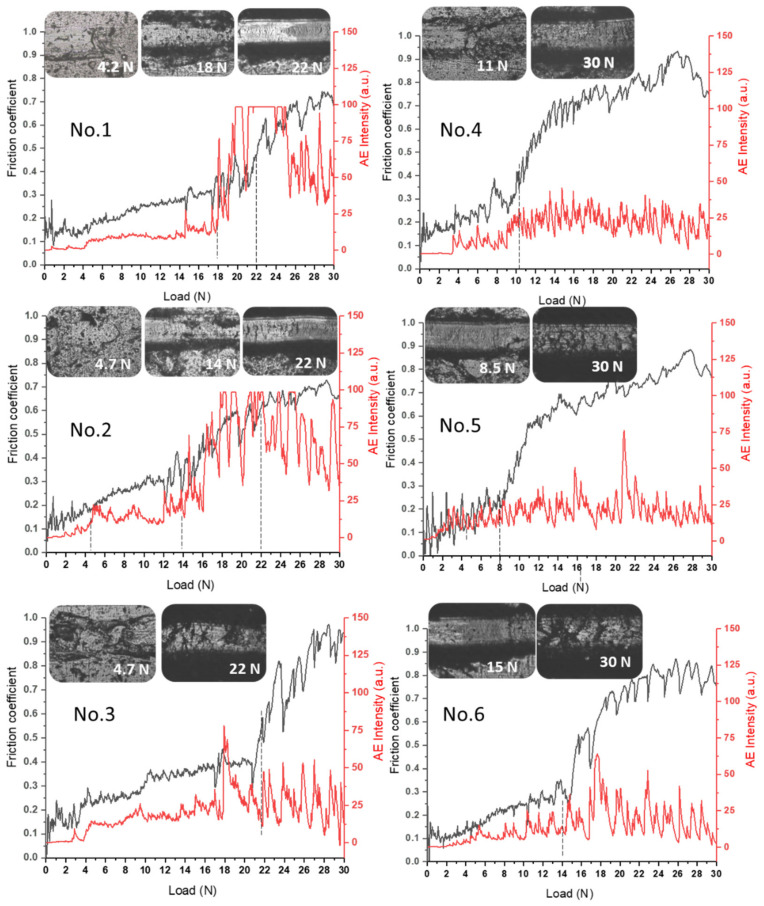
Results of scratch testing of (TiZrNb)N coatings.

Auger electron spectroscopy was used for quantitative analysis of the coatings (Figure 5). The study of the distribution of the coating components (TiZr/Nb)N over the coating thickness indicates that the chemical elements are distributed fairly evenly over the cross-section of the coatings and the distribution curves of the elements have sharp peaks, which does not indicate the interaction between the layers under the deposition conditions, U = −200 V and *p* = 1 × 10^−3^ Torr (samples No. 1, No. 2, No. 3). The presence of iron in the composition of the coatings at a depth of more than 2 μm from the side of the coatings indicates that an increase in the electrical bias provides reliable adhesion of the coating to the steel substrate (Figure 5, samples No. 4 and No. 5), which is confirmed by the results of the testing coatings for adhesive strength (Figure 6). As a result of the analysis of the chemical states of the elements in the (TiZr/Nb)N coatings, it can be noted that it is niobium that makes the main contribution to the composition of the coatings. These results are in good agreement with the result of the X-ray phase analysis (Figure 4).

Based on the results of the distribution of elements over the thickness of the coatings, it is possible to analyze the nature of the formation of the coatings depending on the deposition mode. An increase in nitrogen pressure leads to an increase in the nitrogen concentration in the coating composition, while the content of metal elements decreases (Figure 5 sample No. 5). An increase in the deposition time of each individual layer at U = −100 V and *p* = 4 × 10^−3^ Torr leads to an uneven distribution of elements over the coating thickness and the formation of a relatively wide coating–substrate transition layer (Figure 5, samples No. 4 and No. 5). This can be explained by a shorter relaxation time for the redistribution of metal ions in the diffusion layer during the deposition of each layer. Thus, the modulation in the composition will not occur properly and the characteristics of the coatings approach a monolithic alloy, not a multilayer structure [42]:

Figure 6 shows the results of testing the adhesive strength of (TiZrNb)N coatings by scratch testing. When testing the adhesion strength of coatings, different critical load thresholds can be clearly distinguished. According to the test results of coatings obtained at U = −200 V and *p* = 1 × 10^−3^ Torr, cohesive failure begins at a minimum load (Lc_1_) of 4.2–4.7 N (samples No. 1, No. 2, No. 3). Different coating modes correspond to different values of acoustic emission (AE) depending on the load. The onset of the appearance of the first crack under the indentation load Lc_2_ can be associated with adhesive failure of the coatings (plastic abrasion). At load values Lc_2_ = 18 N and Lc_2_ = 14 N, multiple delaminations of the coating were observed at the edges of the scratches (Figure 3, samples No. 1, No. 2), which correlates with a sharp increase in AE intensity. Comparative analysis shows that the coatings wear out during scratching but do not exfoliate and are destroyed by the cohesive mechanism associated with plastic deformation and the formation of fatigue cracks in the coating material. The critical load of cohesive–adhesive failure Lc_3_ is reached at 22 N (Figure 6, samples No. 1, No. 2). The coating obtained with a deposition time of each layer of 40 s has a good adhesive property when reaching the maximum possible normal load (30 N) on the scratch tester and did not experience destruction (splits, delaminations), which is clearly seen in the image of the track left by the diamond indenter on the coating (Figure 6, sample No. 3), and the AE signal level does not undergo a characteristic jump observed during coating spalling (Figure 6, sample No. 1). An increase in the friction coefficient with increasing load may be due to the degradation of coatings, which leads to the formation of wear products in the form of particles consisting of solid nitrides and leads to the abrasion of the coating (Figure 6, sample No. 3). Similar results were obtained for the (TiZrNb)N coatings at U = −100 V; *p* = 4 × 10^−3^ Torr, which showed high adhesive strength and some degree of delamination in the scratch area at the maximum test load (Figure 6, samples No. 4, No. 5, No. 6). The results were very similar to those reported in [43].

**Figure 7:**
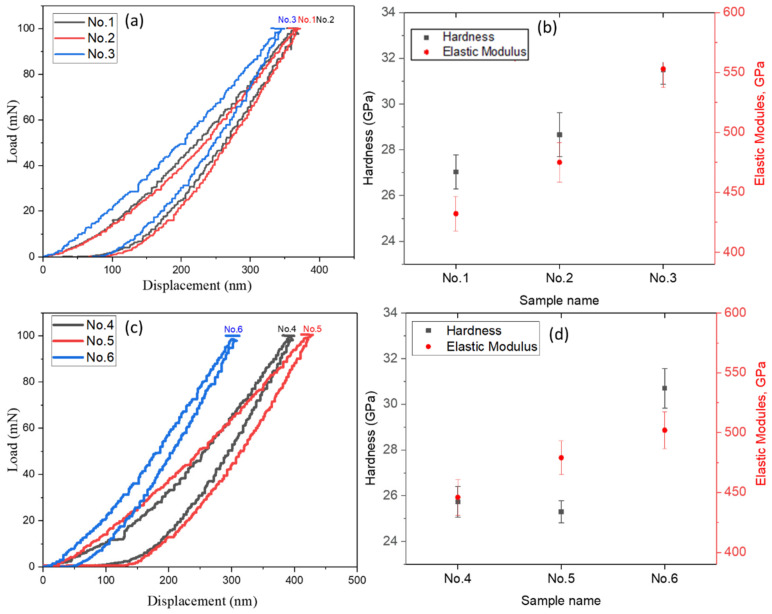
Results of nanoindentation of (TiZrNb)N coatings: (**a**–**c**) Typical indentation force–penetration curve during nanoindentation test; (**b**,**d**) The hardness and elastic modulus.

Figure 7 shows the results of a study of the modulus of elasticity and hardness of multilayer coatings with different numbers of layers. It was revealed that high values of hardness, degree of elastic recovery, and adhesive characteristics are characteristic of the (TiZrNb)N coatings obtained with an individual layer deposition time of 40 s for both investigated modes of vacuum arc sputtering (Figure 7b, samples No. 3 and No. 6). The (TiZrNb)N multilayer coating with more layers (less deposition time for each individual layer) shows the lowest hardness value compared to coatings obtained with an individual layer deposition time of 40 s (number of layers 134). The layered structure of thin films is one of the most developed strategies for obtaining coatings for high-load conditions [44]. Due to the pinning of dislocations at the interfaces of the layered structure, i.e., the barrier effect of interfaces for the movement of dislocations, these materials are characterized by high hardness when applied in the form of a multilayer structure. However, the results of this study showed that an increase in the number of layers does not lead to an increase in the hardness of the coatings with a layered structure. It was also found in [45] that an increase in the number of layers improved the microhardness of multilayer layers up to 128 layers, and then the microhardness decreased. Thus, based on the results of nanoindentation, it can be stated that with an increase in the number of layers, the characteristics of the coatings approach a monolithic alloy rather than a multilayer structure.

To resist abrasive and adhesive wear, coatings must have high hardness and a high value of elastic recovery, which is especially important under conditions of impact, abrasive and erosive effects. Such a value as H/E, which characterizes the stability of a material to elastic fracture deformation and is called the index of plasticity, can be used to assess the wear resistance of coatings [41]. The plasticity index H/E for superhard coatings should be more than 0.1 [42,43,44]. To do this, a coating with high hardness (H) must have a relatively low elastic modulus (E). However, for all studied (TiZrNb)N coatings, H/E values were determined to be around 0.05–0.06.

**Figure 8:**
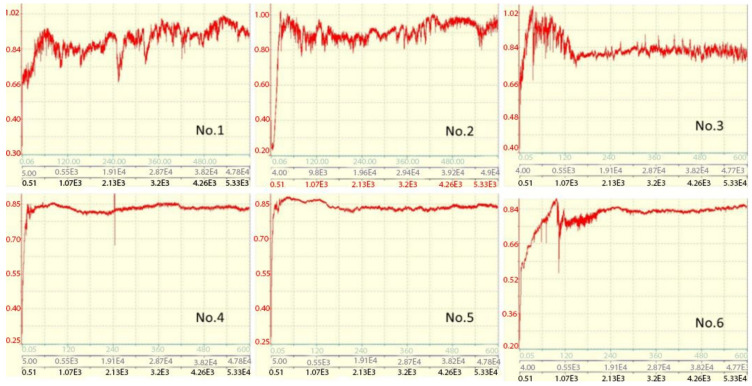
Dependence of the friction coefficient of (TiZrNb)N coatings on the distance, number of cycles and experiment time.

**Figure 9:**
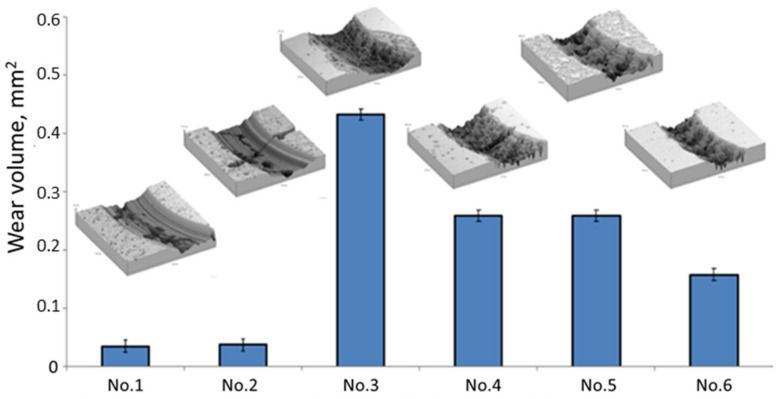
The wear volume of (TiZrNb)N coatings with the transverse profile of the wear track.

Tribological properties, such as friction coefficient and wear coefficient, are important parameters that determine the performance of coatings. The friction coefficient determines the bonding strength of the rubbing materials and the abrasion resistance according to the wear volume, that is, the less the wear, the higher the abrasion resistance. The wear volume of the (TiZrNb)N coatings was calculated from the cross-sectional profile area of the wear track. The results of the tribological studies (Figure 8) showed that the (TiZrNb)N coatings have a high coefficient of friction, the average value of which is equal to μ = 0.85–0.95 (Figure 8). Such values can be explained by the high roughness of the wear tracks due to the cohesive destruction of the coatings, in accordance with the profilogram of the wear tracks (Figure 9). The reasons for the reduced serviceability of the multilayer coatings of (TiZr/Nb)N, obtained by the vacuum arc method, which in this study demonstrated the highest antifriction properties, remain unclear. For their elucidation, in our opinion, additional studies of the peculiarities of contact and micro-destruction processes of coatings under conditions of heavily loaded frictional contact are necessary. In particular, a special study should be performed considering the nature of processes of the formation of surface damage, arising in such conditions and resulting in the development of intensive fluctuations of friction force and instability (f). It is unequivocal to say that these fluctuations and the instability of the friction coefficient are connected exactly with the catastrophic destruction of the coating, as is usually carried out [46,47], as any appreciable traces of such destruction on the friction tracks received after tribological tests are present (Figure 9).

## 4. Conclusions

The phase composition, elemental structure and mechanical and tribological properties of multilayer nanocoatings (TiZr/Nb)N obtained by the Arc-PVD method were investigated. The main results of the work are:

- It was established that the (TiZr/Nb)N multilayer coating consists of a solid solution of (TiNb)N and ε-NbN with an FCC structure. Based on the phase composition data, it can be assumed that an increase in the number of bilayers leads to a decrease in the concentration of nitrogen in the bilayers and, consequently, to a decrease in the volume fraction of ε-NbN and β-Nb_2_N nitrides and the appearance of a weak peak of the solid solution of (TiZr)N;
- It was determined that an increase in the bias potential from −100 V to −200 V leads to an increase in the coating thickness (TiZr/Nb)N and is from 8–10 µm to 10–20 µm, respectively. It was also found that an increase in the deposition time of each individual layer at U = −100 V and *p* = 4 × 10^−3^ Torr leads to an uneven distribution of elements over the coating thickness and the formation of a relatively wide coating–substrate transition layer. Due to the shorter relaxation time for redistribution of metal ions in the diffusion layer during the deposition of each layer, the modulation of elements in the composition will not be ideal, and the characteristics of the coatings are close to a monolithic alloy, not multilayer structure;
- It was determined that the multilayer coating (TiZr/Nb)N has a high adhesion strength when tested with abrasion fracture using the cohesive mechanism. Cohesive destruction of coatings leads to a decrease in the wear resistance of the multilayer coatings;
- The possibility of controlling the mechanical and tribological properties of multilayer (TiZrNb)N coatings by Arc-PVD was shown. The regularities of the formation of the structural-phase state and the features of the change in the mechanical and tribological characteristics of multilayer Arc-PVD (TiZr/Nb)N coatings were established depending on the pressure of the reaction gas and the number of bilayers.

## Figures and Tables

**Table 1:** Deposition conditions for (TiZr/Nb)N coatings using the Arc-PVD method.

Sample	Id, A	I_f_, A	U	P, Torr	Notes	Time
No. 1	100	0.5	200	1 × 10^−3^	Interval 10 s 536 layers	1.5 h
	80	0.5				
No. 2	100	0.5	200	1 × 10^−3^	Interval 20 s 270 layers	1.5 h
	80	0.5				
No. 3	100	0.5	200	1 × 10^−3^	Interval 40 s 134 layers	1.5 h
	80	0.5				
No. 4	100	0.5	100	4 × 10^−3^	Interval 10 s 536 layers	1.5 h
	80	0.5				
No. 5	100	0.5	100	4 × 10^−3^	Interval 20 s 270 layers	1.5 h
	80	0.5				
No. 6	100	0.5	100	4 × 10^−3^	Interval 40 s 134 layers	1.5 h
	80	0.5				

Id, A–current on cathodes, I_f_, A–discharge current to clean the surface before deposition, U–substrate potential, P, Torr–Nitrogen reaction gas operating pressure.

**Table 2:** Results of X-ray study of multilayer coatings (TiZr/Nb)N.

Sample	Detected Phases	CSR Size, nm	Δd/d × 10^−3^
No. 1	TiN	12	0.8
	NbN	18	7.7
	ZrN	10	6.4
	Nb_2_N	16	1.8
	Ti_2_N	15	2.1
No. 2	TiN	15	0.3
	NbN	20	7.
	ZrN	9	5.8
	Nb_2_N	13	2.6
	Ti_2_N	16	2.6
No. 3	TiN	16	0.8
	NbN	20	7.4
	ZrN	9	5.4
	Nb_2_N	16	2.0
	Ti_2_N	24	3.5
No. 4	TiN	10	4.7
	NbN	11	5.0
	ZrN	12	5.1
	Nb_2_N	13	7.1
No. 5	TiN	11	3.6
	NbN	12	6.3
No. 6	TiN	14	1.8
	NbN	11	6.0

## Data Availability

Not applicable.
